# U-shaped stereoscopic design strategy toward N-doped nanographene segment†

**DOI:** 10.1039/d4ra00788c

**Published:** 2024-04-12

**Authors:** Dongyang Zhang, Fengyuan Zhang, Hanyun Du, Fei Liu, Xiaohui Yi, Jun Chen, Ben-Lin Hu

**Affiliations:** a School of Materials Science and Engineering, Jiangxi Provincial Key Laboratory of Power Batteries and Materials, Jiangxi University of Sciences and Technology Ganzhou 341000 China chenjun@jxust.edu.cn; b CAS Key Laboratory of Magnetic Materials and Devices, Zhejiang Province Key Laboratory of Magnetic Materials and Application Technology, Ningbo Institute of Materials Technology and Engineering, Chinese Academy of Sciences Ningbo 315201 China zhangfengyuan@nimte.ac.cn yixiaohui@nimte.ac.cn hubenlin@nimte.ac.cn; c School of Materials Science and Chemical Engineering, Ningbo University Ningbo 315211 China

## Abstract

There have been scarce reports about stereoscopic design of N-heteroacenes (NHAs), especially for the electron-deficient π-building blocks. Herein, we report the design and synthesis of a U-shaped bis(pyrene-quinoxaline) (BPQ). Single crystal X-ray diffraction reveals the herringbone stacking pattern and the presence of regular and incompletely closed pores.

## Abstract 2

Through a stereoscopic design strategy, U-shaped N-heteroacene was synthesized, and shows regular and incompletely closed pores within the crystal structure.

## Graphical abstract

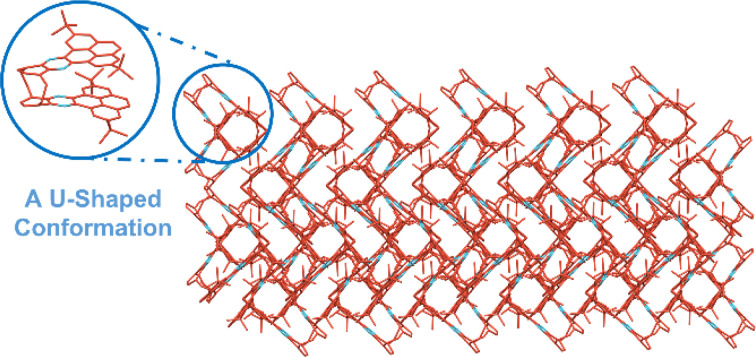

N-Heteroacenes (NHAs), through the substitution of nitrogen atoms for C–H units, are frequently utilized as electron-deficient π-building blocks, constructing indispensable structural elements in N-doped nanographenes.^1,2^ Due to the presence of lone pairs of electrons and the higher electronegativity of nitrogen atoms, their incorporation significantly alters the photoelectric and physicochemical properties of the acenes and arenes. Nitrogen doping typically leaves the planarity of the acenes and arenes unaffected but leads to finely tuneable frontier molecular orbital, enhancing electron affinity and oxidative stability. Since the synthesis of the first N-heteroacene in the 1960s, considerable efforts have been devoted to their potential applications in organic optoelectronic devices, spanning from flexible displays, electronic skin, wireless radio frequency identification tags, and organic thermoelectrics.^3–5^ With their unique photophysical and electronic properties, NHAs are esteemed as essential components of heterographene, and their fundamental structural and topological features have formed the crux of research in the field of heteroatom-doped organic optoelectronics.^6,7^ For instance, tetraazapentacenes (TAPs), which serve as prototypical n-type channel organic semiconductors for optoelectronic applications, have garnered significant attention. Recently, the linear molecular lengths of B-, S- and O-doped acenes have been successfully expanded beyond nine, revealing their significant potential in organic electronics.^8–10^ However, molecular design has predominantly been confined to one-dimensional linear structures, while heteroatom-doped polycyclic aromatics with stereoscopic design remain elusive.

Among the several strategies, adjusting structural parameters, including edge structure, width, length, and heteroatom-doping, has proven to be effective in enhancing stability and optimizing optoelectronic and magnetic properties.^11^ Recognizing their critical role in both theories and experiments, considerable research efforts have been devoted to the development of pertinent heteroaromatics. Currently, research on NHAs primarily focuses on designing and incorporating electron-withdrawing groups, such as fluorine, chlorine, imide, or cyano groups. Alternatively, studies also explore the elongation of NHA chains.^2,6,12^ In 2018, the 4Cl-tetraazapentacene (4Cl-TAP) reported by Miao *et al.* exhibited an impressive electron mobility of 27.8 cm^2^ V^−1^ s^−1^, marking an important milestone in the field of n-type organic field-effect transistors.^13^ The synthesis of longer NHAs faces significant challenges due to their low solubility in organic solvents and instability under ambient conditions. NHA long chains comprising 18, 30, and even 60 rings have been successfully designed and synthesized.^11,14,15^ However, there have been comparatively limited reports on stereo-structure design of NHAs, especially for electron-deficient π-building blocks. In 2019, Hu *et al.* reported three-dimensional NHAs with an impressive diameter of up to 10.88 nm, demonstrating the effective strategy of integrating two-dimensional building blocks, such as nanographenes and graphene sheets, into three-dimensional frameworks to extend the size and prevent the aggregation of two-dimensional structures.^16^ In addition, the investigations by Kawashima and colleagues, as well as Kroeger *et al.*, into the aromatic and catalytic properties of cyclophanes, provide essential insights into the effects of large π-plane proximity.^17,18^ Investigating the structural diversity of NHAs and their micromorphology in the solid state will contribute to a more profound understanding of their novel pathways as electronic materials.

The 2,1,3-benzothiadiazole (BTD) is known for its superior electron affinity and is frequently utilized as an electron acceptor, particularly in the realm of electron transport materials. As shown in Scheme 1, BTD dimer forms a unique spatial configuration through bridging carbon connections.^19^ Among the strategies adopted for the construction of these stable, elongated NHAs, the introduction of pyrene is most prevalent. Herein, we report on the design, synthesis, and properties of bis(pyrene-quinoxaline) (BPQ). BPQ, a N-doped pyrene–naphthalene derivative, generated by the condensation of tetra-amine, a reduction product of a 2,1,3-benzothiadiazole dimer, with diketone. The BPQ possesses a U-shaped configuration, in which the bilayered (*N* = 4 +4) fused-ring units are tightly connected by bridging carbon atoms that serve to reinforce the π-bonding interactions (DFT calculations; see Fig. S1 and S2 in the ESI†).

**Scheme 1:**
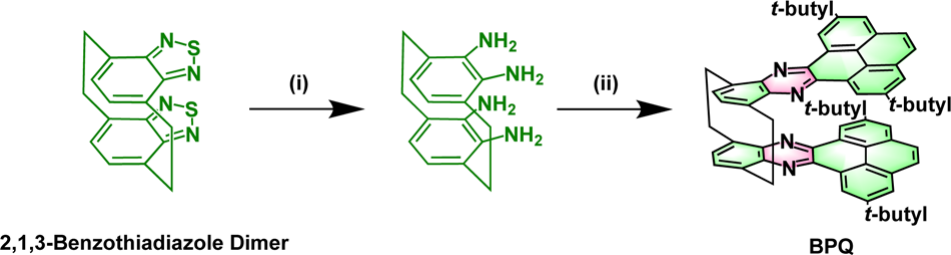
Synthesis of BPQ. Reagents and conditions: (i) LiAlH_4_/THF/60 °C/12 h; (ii) 2,7-di-*tert*-butylpyrene-4,5-dione/acetic acid/110 °C/24 h.

The synthesis of BPQ is depicted in Scheme 1. The intermediate tetra-amine was obtained almost quantitatively by the reduction of BTD dimer with LiAlH_4_ in anhydrous THF (detailed steps in Scheme S1 and the NMR spectra in Fig. S3–S6†). Diketone was obtained by oxidizing 2,7-di-*tert*-butylpyrene in a mixture of dichloromethane and acetonitrile, which was facilitated by RuCl_3_·*x*H_2_O as a catalyst, and synthesized according to the reported procedures.^20^BPQ was successfully synthesized by the condensation under reflux in an acetic acid solution, which demonstrated remarkable solubility in common organic solvents such as DCM, THF and CHCl_3_. The ^1^H NMR spectrum clearly revealed two distinct sets of methylene hydrogen signals, attributing to the hydrogen atoms on the bridge carbons (Fig. S7†), primarily due to the rigidity of the structure. The structure of BPQ was unambiguously characterized through attached proton test (APT) ^13^C NMR, 2D ^1^H–^13^C heteronuclear single quantum coherence (HSQC) NMR and liquid chromatography-quadrupole time-of-flight tandem mass spectrometry (LC-Q-TOF-MS) (for detailed synthetic procedures and characterization data, see Scheme S2 and Fig. S8–S11 in the ESI†).

The ultraviolet-visible (UV-Vis) absorption spectrum of BPQ in diluted DCM (10^−6^ M) is shown in Fig. 1a. There are three distinct bands in the spectrum, located at 300–375 nm (first band), 390–420 nm (second band), and 420–510 nm (third band), corresponding to β, ρ and α bands, respectively, which agrees with Clar's nomenclature in the polycyclic aromatic hydrocarbons (PAHs).^21,22^ The first and second bands are attributed to the π–π* transition and n–π* transitions of the conjugated aromatic segments.^23^ In the third band, the peaks of BPQ can be attributed to 0–1 and 0–0 transitions.^24^ The onset absorptions of BPQ is 494 nm and the corresponding energy gaps of approximately 2.51 eV, significantly smaller than that of pyrene (3.59 eV).^25^ The normalized photoluminescence (PL) spectrum of BPQ in dilute DCM (10^−7^ M) is also presented in Fig. 1a. Upon photoexcitation at a wavelength of 400 nm, BPQ manifests a yellow-green fluorescence, consistent with our observations. The peak emission wavelength is at 533 nm, with the Stokes shifts of 136 986 cm^−1^. The quantum efficiency of BPQ in dichloromethane has been determined to be 3.7%. In comparison to the monomeric pyrene-quinoxaline reported by Lindner *et al.*, the onset absorption of BPQ is red-shifted from 473 nm, as observed for the monomer, to 494 nm. This spectral shift corresponds to a decrease in the optical bandgap from 2.62 eV to 2.51 eV. Furthermore, the maximum emission wavelength transitions from 461 nm to 533 nm, which signifies that there are notable differences in the photophysical properties between the monomer and BPQ, particularly in their fluorescence emission characteristics. This clear distinction underscores the impact of molecular architecture on optoelectronic behavior.^26^

**Fig. 1:**
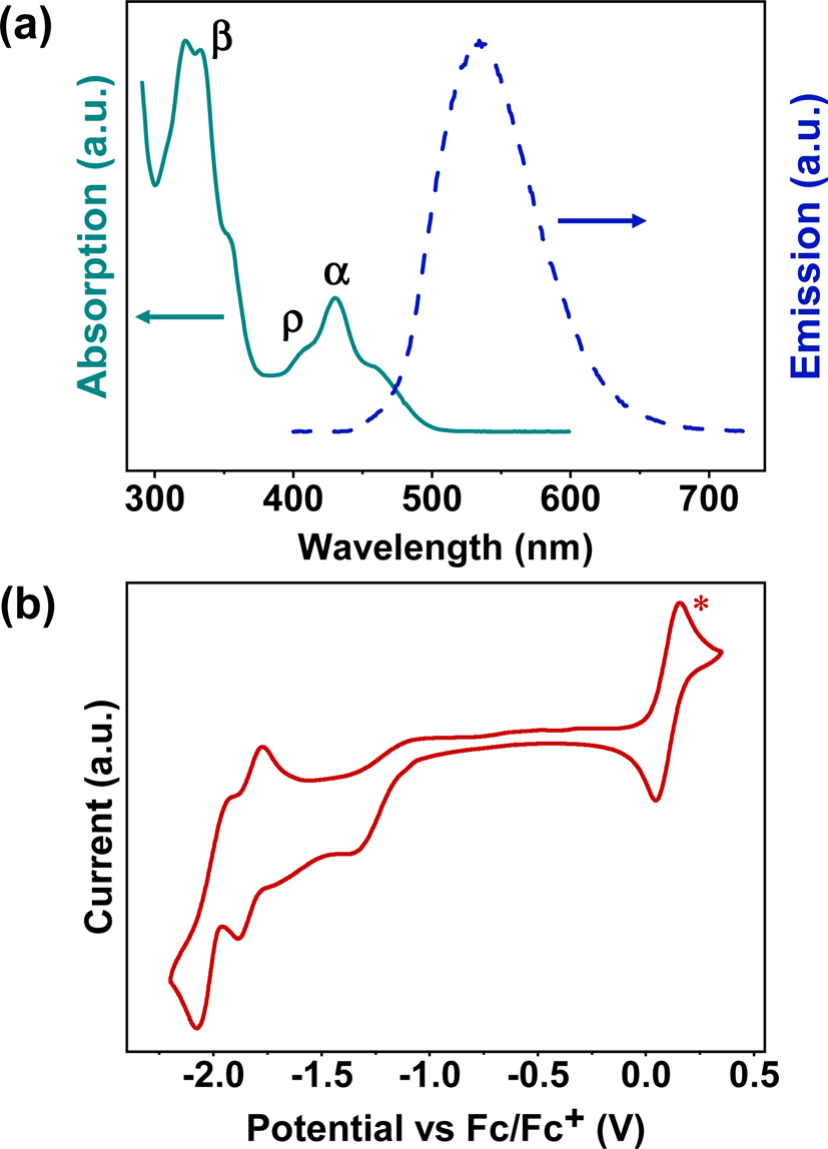
(a) UV-Vis absorption (10^−6^ M, solid line) and fluorescence emission (10^−7^ M, dash line) spectra of BPQ in DCM. (b) Cyclic voltammogram of BPQ in DCM with ferrocene (ferrocene peaks are indicated by red star and occur at positive potential) as an internal standard, the energy of Fc/Fc^+^ was assumed as −4.8 eV relative to vacuum.

The cyclic voltammetry (CV) curve of BPQ in diluted DCM (or THF) with tetrabutylammonium hexafluorophosphate (*n*-Bu_4_PF_6_) as supporting electrolyte (0.1 M) is shown in Fig. 1b and S12.† Furthermore, BPQ exhibits three distinct reduction behaviors with the *E*^red^_1/2_ at −1.13 eV, −1.79 eV and −1.98 eV respectively. Notably, two of these redox processes are quasi-reversible. This is indicative of its excellent electron-accepting capabilities. The onset voltages of the reduction peaks are −1.13 eV and the corresponding electron affinities (EAs) are 3.67 eV. All of these data are summarized in Table 1. Relative to the monomeric pyrene-quinoxaline, the lowest unoccupied molecular orbital (LUMO) level of BPQ is reduced from −3.49 eV to −3.65 eV, and the highest occupied molecular orbital (HOMO) level declines from −6.11 eV to −6.16 eV. This effectively lowers the molecular frontier orbitals, which may confer advantageous electronic properties relevant to organic semiconductor applications.

**Table 1:** Photophysical and electrochemical properties and energy levels of BPQ

Compound	*λ* ^0−1^_abs_ (nm)	*λ* ^0−0^_abs_ (nm)	*λ* ^onset^_abs_ (nm)	*E*_gap_ a (eV)	*λ*_em_ (nm)	*E*_onset,red1_ b (V)	EA^c^ (eV)	*E*_LUMO,DFT_ (eV)	*E*_HOMO,DFT_ (eV)
BPQ	429	460	494	2.51	533	−1.13	3.67	−2.13	−5.30

a Estimated from the absorption onset.

b The first reduction peak, measured in *n*-Bu_4_NPF_6_ solution in CH_2_Cl_2_ with a scan rate of 10 mV s^−1^ and ferrocene as an internal standard.

c Estimated from *E*_onset, red1_; the energy of Fc/Fc^+^ was assumed as −4.8 eV relative to vacuum.^27,28^

Yellow plate-like single crystals of BPQ were acquired through the slow evaporation of DCM under ambient conditions. The quality of the BPQ crystals was sufficient for single-crystal X-ray diffraction analysis. Monoclinic system (*C*2/*c* space group) was found for the BPQ crystals (for the unit cell parameters, see Table S1†).

A bilayer quasi-planar conjugated backbone composed of bridged carbon, was observed for BPQ, as depicted in Fig. 2a (see picture; for clarity, all hydrogen atoms are omitted; C red and N blue). The BPQ adopted a U-shaped configuration, resulting in a dihedral angle of 11.64° (Fig. S13†). This configuration resulted in a slight twist within the conjugated framework, with variation in the N⋯N distances, 3.38 Å and 3.34 Å, respectively, attributing to the structural rigidity. Owing to the interlayer repulsion, the planarity of the pyrene was disrupted. Structurally, BPQ can be conceptualized as a segment of N-doped nanographene (Fig. S14†), exhibiting dimensions of 11.23 Å in length and 2.76 Å in width. Two distinct orientations of BPQ were present within the crystal, consistent with the herringbone packing with π–π overlap (Fig. 2b).^29–31^ Due to the substantial repulsion between the *tert*-butyl units, BPQ exhibited limited π–π stacking with a distance of 3.37 Å (Fig. S15†). Additionally, an observable displacement between the upper and lower acene frameworks containing the pyrene skeleton is recorded, resulting in the formation of a skewed arrangement with a measured offset angle of 26.81° (Fig. S16†). Furthermore, there were also intermolecular C⋯H non-covalent interactions within the herringbone packing, which were located within the van der Waals radius of C⋯H (2.82 Å), forming the intermolecular conformational locks (Fig. 2c).^32–34^ Interestingly, the herringbone packing of U-shaped from different layers (distinguished by yellow and green for varying planes) constructed heart-shaped patterns (Fig. 2d), with these heart-shaped motifs densely packed within the crystal to form regular, albeit incompletely enclosed, voids.

**Fig. 2:**
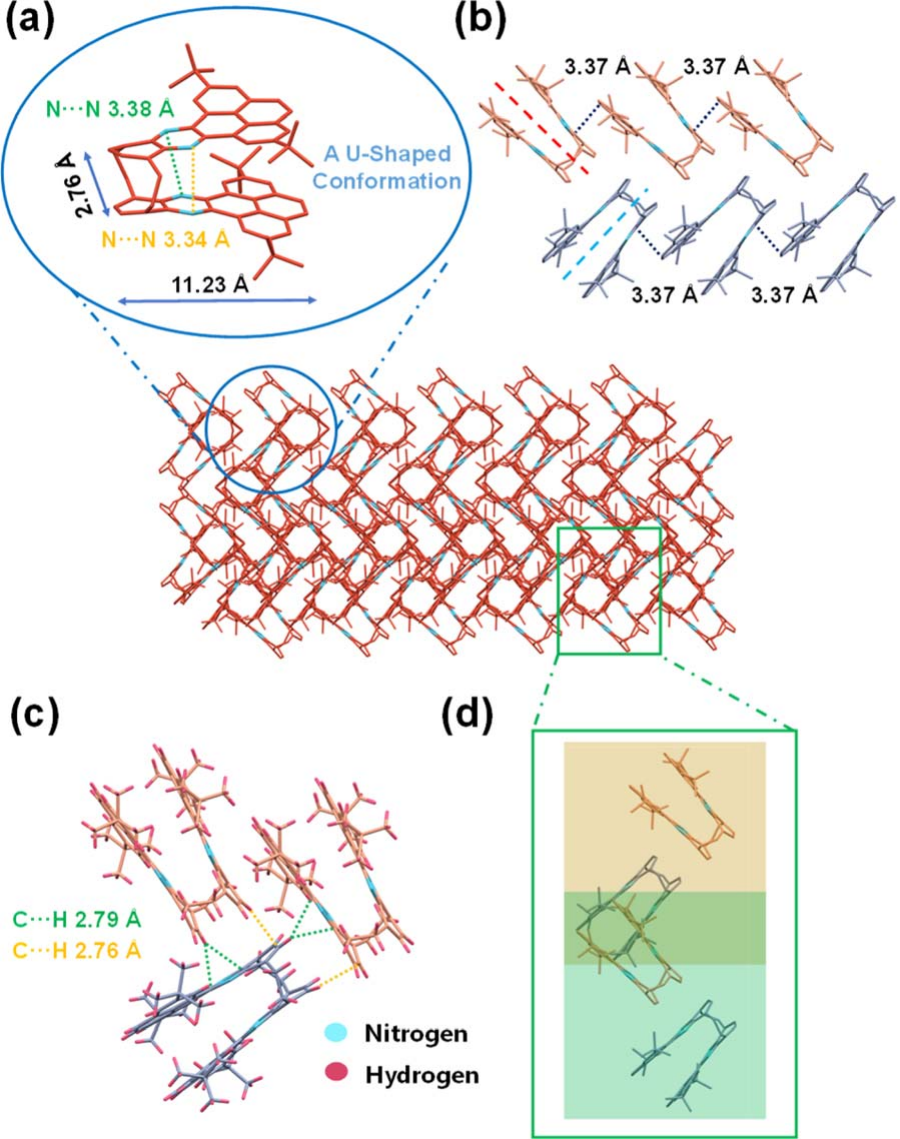
Crystalline configuration and aggregation pattern of BPQ: (a) U-shaped configuration, (b) the herringbone packing with π–π overlap; (c) C⋯H non-covalent bond interactions and (d) the herringbone packing of the different layers (represented by yellow and green) constructed heart-shaped patterns.

In conclusion, bis(pyrene-quinoxaline) (BPQ) has been synthesized employing the stereoscopic design strategy, endowing it with a distinct U-shaped configuration. The molecular structure features bilayered fused-ring units (*N* = 4 + 4). Single crystal X-ray diffraction reveals a herringbone stacking motif, accompanied by the formation of regular and incompletely closed pores. This design strategy not only allows for precise control over the nitrogen doping sites and stereo configuration but also significantly enhances the control over the spatial arrangement of nanographene segments. Taking advantage of the N-doping in acenes and arenes, these structures show potential for application in organic electronics and energy conversion, inspiring the design of larger two-dimensional or even three-dimensional NHAs based on the stereo-structured electron-deficient π-building blocks.

## Conflicts of interest

There are no conflicts to declare.

## Supplementary Material

RA-014-D4RA00788C-s001

RA-014-D4RA00788C-s002
