# Supplementary material for: U-shaped stereoscopic design strategy toward N-doped nanographene segment
Source: RSC Adv. 2024 Apr 12;14(17):11771–4. doi: 10.1039/d4ra00788c (PMC11009840; doi:10.1039/d4ra00788c)
Supplement: RA-014-D4RA00788C-s001 [file RA-014-D4RA00788C-s001.pdf]

Electronic Supplementary Material (ESI) for RSC Advances.
This journal is © The Royal Society of Chemistry 2024

Supporting Information

U-Shaped Stereoscopic Design Strategy Toward N-Doped Nanographene Segment

Dongyang Zhang,[ab] Fengyuan Zhang,*[b] Hanyun Du,[bc] Fei Liu,[b] Xiaohui Yi,*[b] Jun Chen,*[a] and Ben-Lin Hu*[b]

a. School of Materials Science and Engineering, Jiangxi Provincial Key Laboratory of Power Batteries and Materials, Jiangxi University of Sciences and Technology, Ganzhou 341000, China.

b. CAS Key Laboratory of Magnetic Materials and Devices, and Zhejiang Province Key Laboratory of Magnetic Materials and Application Technology, Ningbo Institute of Materials Technology and Engineering, Chinese Academy of Sciences, Ningbo 315201, China.

c. School of Materials Science and Chemical Engineering, Ningbo University, Ningbo 315211, China.

Email: zhangfengyuan@nimte.ac.cn; yixiaohui@nimte.ac.cn; chenjun@jxust.edu.cn; hubenlin@nimte.ac.cn

Supporting Information

Contents

Instrumentation 2

Reagents and Synthesis 2

DFT Results 5

NMR Spectra..7

HRMS Spectra 10

Electrochemical Measurement 11

Single Crystal X-Ray Analysis 12

References 14

Instrumentation

Nuclear magnetic resonance (NMR) spectra were recorded on a Bruker AVANCE III 400, Bruker AVANCE NEO 600 spectrometer in $CDCl_3$. Chemical shifts were reported in parts per million (ppm) and referenced to residual $CHCl_3$ (7.26 ppm) for ^1H NMR, and to $CDCl_3$ (77.16 ppm) for ^{13}C NMR. The following abbreviations were used for multiplicities: s = singlet, d = doublet, dd = double of doublets, t = triplet, m = multiplet. High resolution mass spectrometry (HRMS) was performed via atmospheric pressure chemical ionization (APCI) on an AB Sciex liquid chromatography-quadrupole time of flight tandem mass spectrometry (LC-Q-TOF-MS) with dichloromethane (DCM) as the solvent. Ultraviolet–visible (UV-Vis) absorption spectra were measured on a Perkin-Elmer Lambda 950 spectrophotometer at room temperature. Photoluminescence (PL) spectra were recorded on a Horiba FL3-111 spectrofluorometer. The electrochemical measurements were carried out in anhydrous DCM containing 0.1 M n-Bu_4NPF_6 as supporting electrolyte (scan rate: 10 mV s^{-1}) under argon atmosphere on a CHI600E electrochemical analyzer. A three-electrode system with glassy carbon as the working electrode, platinum wire as the reference electrode, another platinum wire as counter electrode was applied. The potential was calibrated against ferrocene/ferrocenium couple. Density functional theory (DFT) calculations were performed using the Gaussian 16 program[1] with the B3LYP hybrid functional[2,3] and basis set 6-31G(d) for the ground state geometry optimization. The single crystals of **BPQ** were obtained by slow evaporation of DCM in solution with concentration of ~0.5 mg/mL in NMR tube. X-ray crystallographic data for the molecules were collected on a Bruker D8 VENTURE dual wavelength Mo/Cu diffractometer. The crystal was kept at 194.0 K during data collection. Using Olex2[4], the structure was solved with the SHELXT[5] structure solution program using Intrinsic Phasing and refined with the SHELXL[5] refinement package using Least Squares minimisation. All the hydrogen atoms were omitted for clarity.

Reagents and Synthesis

2,7-di-*tert*-butylpyrene-4,5-dione[6] and syn-[2.2](4,7)Benzothiadiazolophane **M2**[7] were prepared according to the reported procedures. All starting chemicals, unless otherwise specified, were purchased from Alfa Aesar or Sigma-Aldrich and used as received.

Solvents were purified by normal procedure before use. The other materials were common commercial level and used as received.

Scheme S1. The synthesis of M3

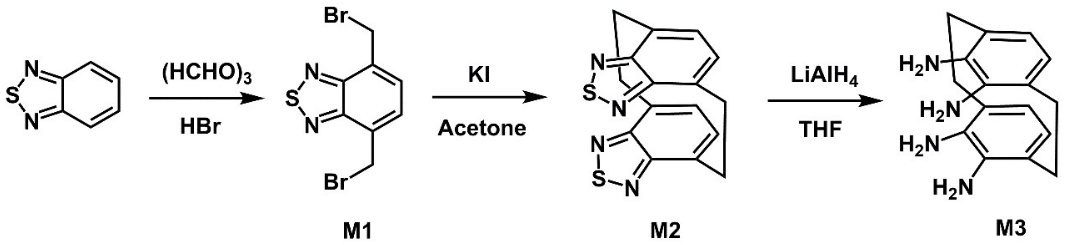

4,7-bis(bromomethyl)benzo[c][1,2,5]thiadiazole (**M1**)

To a 1-neck, 250 mL round bottom flask equipped with a magnetic stir bar and condenser was added 2,1,3-benzothiadiazole (5.00 g, 36.7 mmol), hydrobromic acid (48 wt% in water, 100 mL). 1,3,5-Trioxane (16.6 g, 184 mmol) and tetra-*n*-octylammonium bromide (2.00 g, 3.66 mmol) were added to the flask while stirring the mixture. The reaction was heated to reflux and stirred for 16 hours. The reaction was cooled to room temperature and the precipitate was filtered, washed with water then ethanol and dried under reduced pressure. The resulting off-white solid was further purified using silica gel column chromatography (eluent: dichloromethane/hexanes = 1:3, v/v), yielding **M1** as a white crystalline solid (10.9 g, 92%). ^{1}H NMR (400 MHz, $CDCl_3$): δppm = 7.63 (s, 2H), 4.97 (s, 4H).

syn-[2.2](4,7)Benzothiadiazolophane (**M2**)

A mixture of 4,7-bis(bromomethyl)benzo[*c*][1,2,5]thiadiazole (**M1**) (322 mg, 1.00 mmol), KI (1.66 g, 10.0 mmol), and acetone (25 mL) was refluxed for 18 h with stirring. After cooling, the solvent was removed under reduced pressure and CH_2Cl_2 (25 mL) was added to the residue. The resulting precipitate was filtered and the filtrate was concentrated under reduced pressure. The concentrate was separated by silica gel column chromatography (eluent: dichloromethane/hexanes = 1:3, v/v) to give syn-isomer **M2** as yellow crystal (14.3 mg, 9%). ^{1}H NMR (400 MHz, $CDCl_3$): δppm = 6.80 (s, 4H), 4.16 (dd, 4H, J_1 = 13.16 Hz, J_2 = 4.08 Hz), 3.32 (dd, 4H, J_1 = 13.16 Hz, J_2 = 4.08 Hz).

M3

syn-[2.2](4,7)Benzothiadiazolophane (**M2**) (32.4 mg, 0.100 mmol) was dissolved in deaerated THF (20 mL) under an argon atmosphere and stirred at 0 °C. Then $LiAlH_4$ (1.00 mL, 1.0 M in THF, 1.00 mmol) was slowly added. The mixture was heated to 60 °C for overnight. After cooling to room temperature, the reaction mixture was quenched with aqueous NaOH solution. The above solution was filtered through a pad of Celite and the filtrate was extracted with DCM (10 mL × 3). The combined organic layers were collected and dried over anhydrous $MgSO_4$ (s), filtered, and concentrated to dryness. **M3** was used directly for the next step without further purification. Mp > 300 °C. ^{1}H NMR (400 MHz, $CDCl_3$): δppm = 6.06 (s, 4H), 3.37 (dd, 4H, J_1 = 13.25 Hz, J_2 = 4.09 Hz), 2.89 (dd, 4H, J_1 = 13.25 Hz, J_2 = 4.09 Hz). ^{13}C NMR($CDCl_3$, 600 MHz): δppm = 157.30, 132.19, 128.31, 30.75. HRMS(APCI): *m/z* calcd for $C_{16}H_{21}N_4$ $[M+H]^+$, 269.1766, found 269.1765.

Scheme S2. The synthesis of BPQ

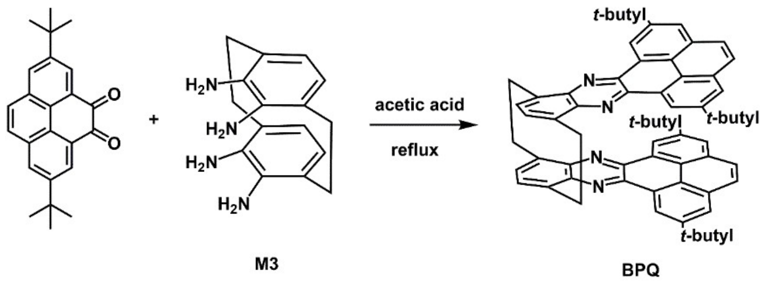

BPQ

2,7-di-*tert*-butylpyrene-4,5-dione (344 mg, 1.00 mmol) and **M3** (134 mg, 0.500 mmol) were dissolved in acetic acid (20 mL), and the mixture was heated to reflux under argon for 24 h. After the mixture cooling to room temperature, all the solvents were removed under vacuum. The residues were elute by silica gel column chromatography (eluent: dichloromethane/hexanes = 1:3, v/v, R_f = 0.35, yellow fluorescence) to obtain the **BPQ** as bright yellow powder (131 mg, 30%). Mp > 300 °C. ^{1}H NMR (600 MHz, $CDCl_3$): δppm = 8.96 (s, 4H), 7.73 (s, 4H), 7.55 (s, 4H), 7.22 (s, 4H), 4.85 (d, 4H, J = 6.24 Hz), 3.39 (d, 4H, J = 6.24 Hz), 1.39 (s, 36H). Attached proton test (APT) ^{13}C ($CDCl_3$, 600 MHz): δppm = 148.19, 142.54, 140.72, 138.72, 132.87, 130.26, 128.28, 126.40, 125.16, 123.31, 120.23, 35.11, 31.86, 31.73. HRMS(APCI): *m/z* calcd for $C_{64}H_{61}N_4$ $[M+H]^+$, 885.4896, found 885.4895.

DFT Results

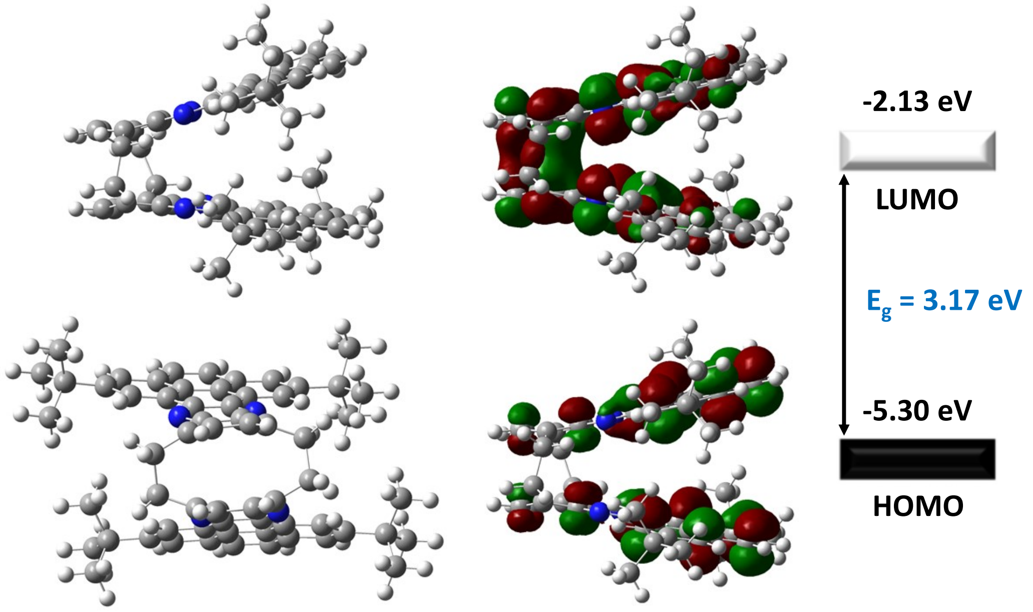

Fig. S1 DFT calculated molecular orbitals and energy diagrams of BPQ at the B3LYP/6- 31G(d) level (the LUMO and HOMO: the lowest unoccupied and highest occupied molecular orbitals).

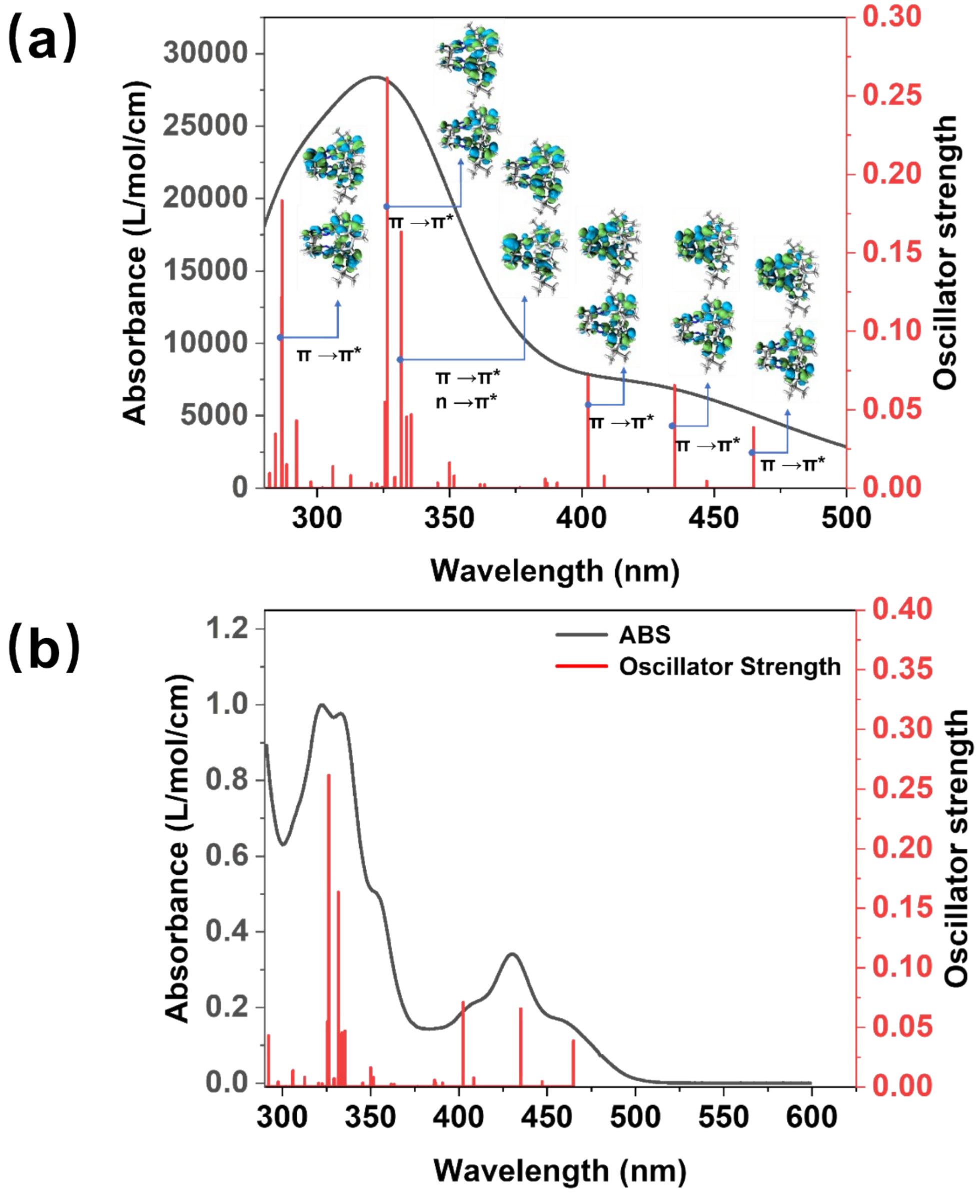

Fig. S2 TDDFT-calculated absorption spectra of BPQ along with the oscillator strengths. (a) The calculated results of the UV-visible absorption spectrum fitted with corresponding energy level transitions. (b) Comparison between the calculated results and the experimental UV-visible absorption spectrum.

NMR Spectra

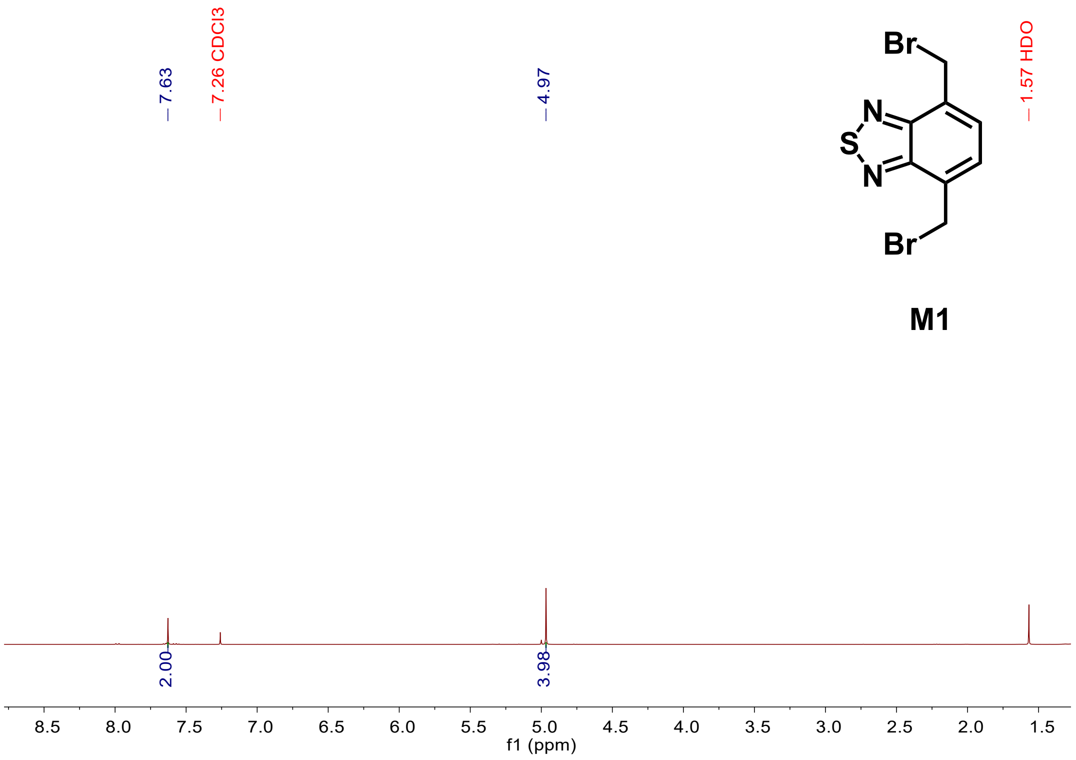

Fig. S3 The ^{1}H NMR spectrum of compound M1 in $CDCl_3$ on 400 MHz.

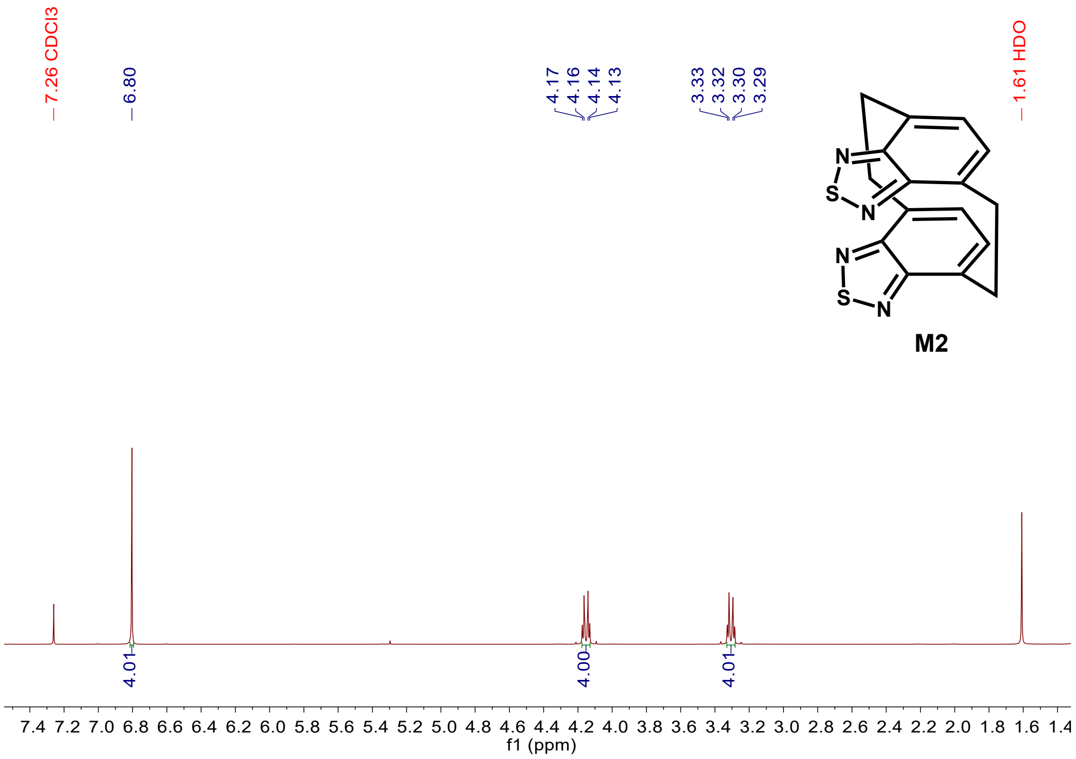

Fig. S4 The ^{1}H NMR spectrum of compound M2 in $CDCl_3$ on 400 MHz.

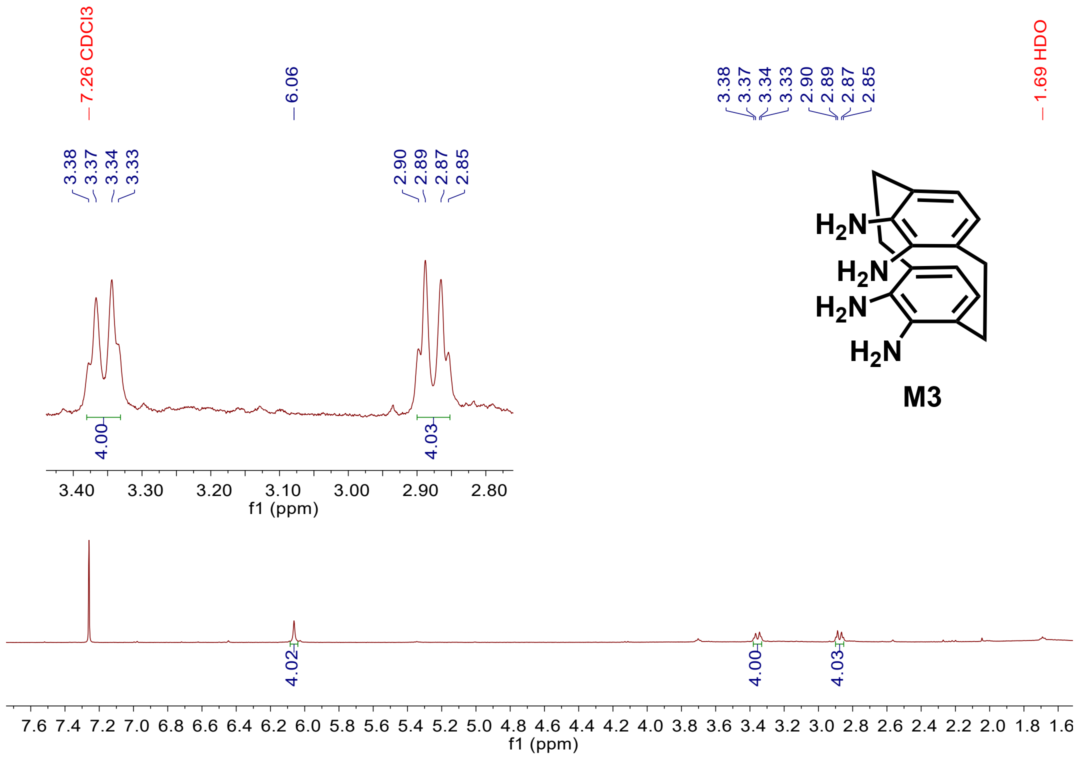

Fig. S5 The ^{1}H NMR spectrum of compound M3 in $CDCl_3$ on 400 MHz.

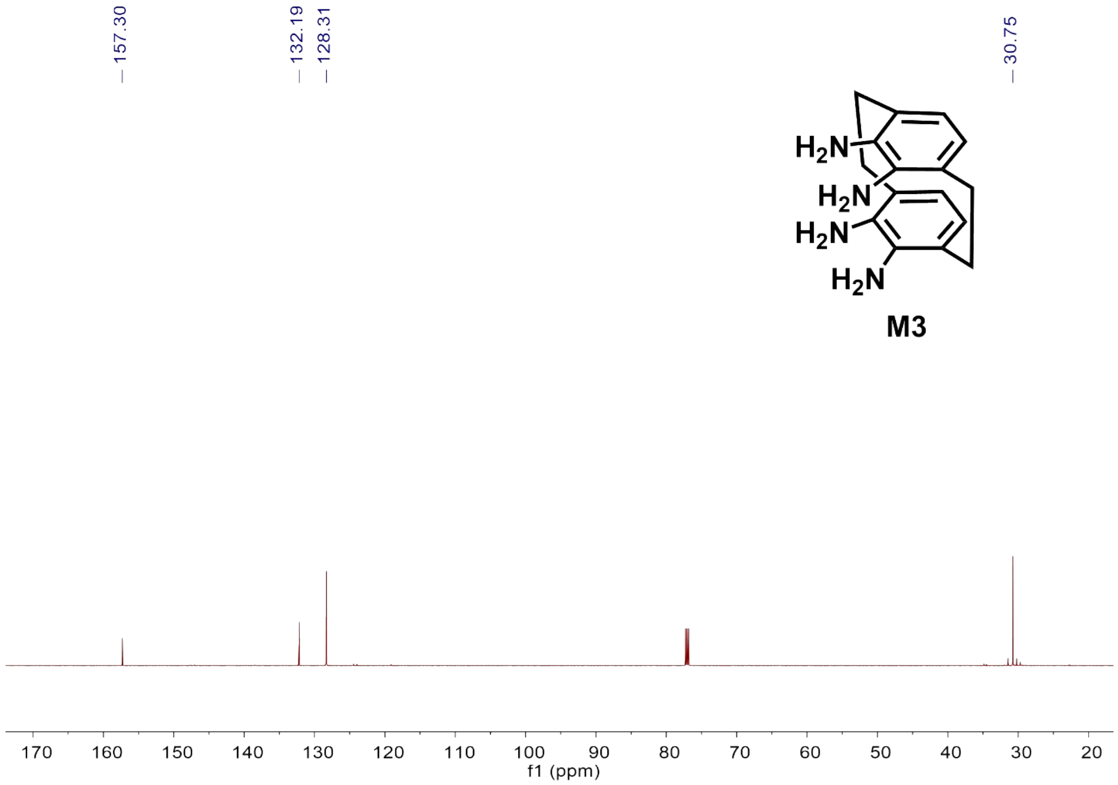

Fig. S6 The ^{13}C NMR spectrum of compound M3 in $CDCl_3$ on 600 MHz.

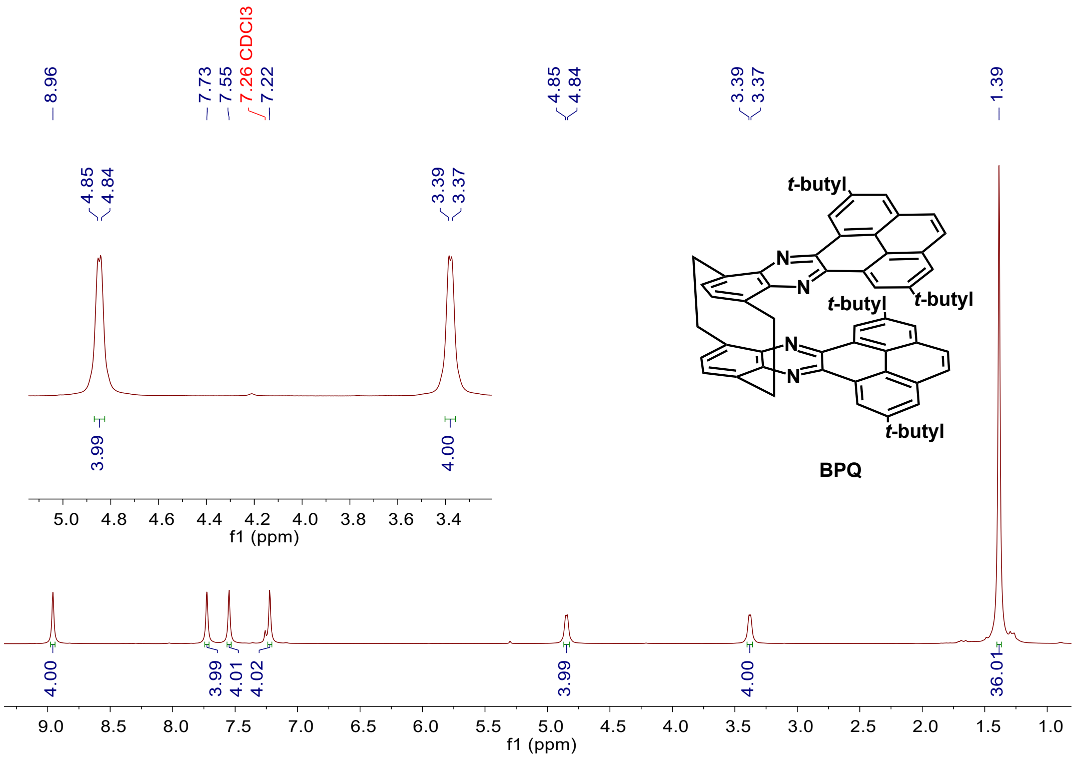

Fig. S7 The ^{1}H NMR spectrum of compound BPQ in $CDCl_3$ on 600 MHz.

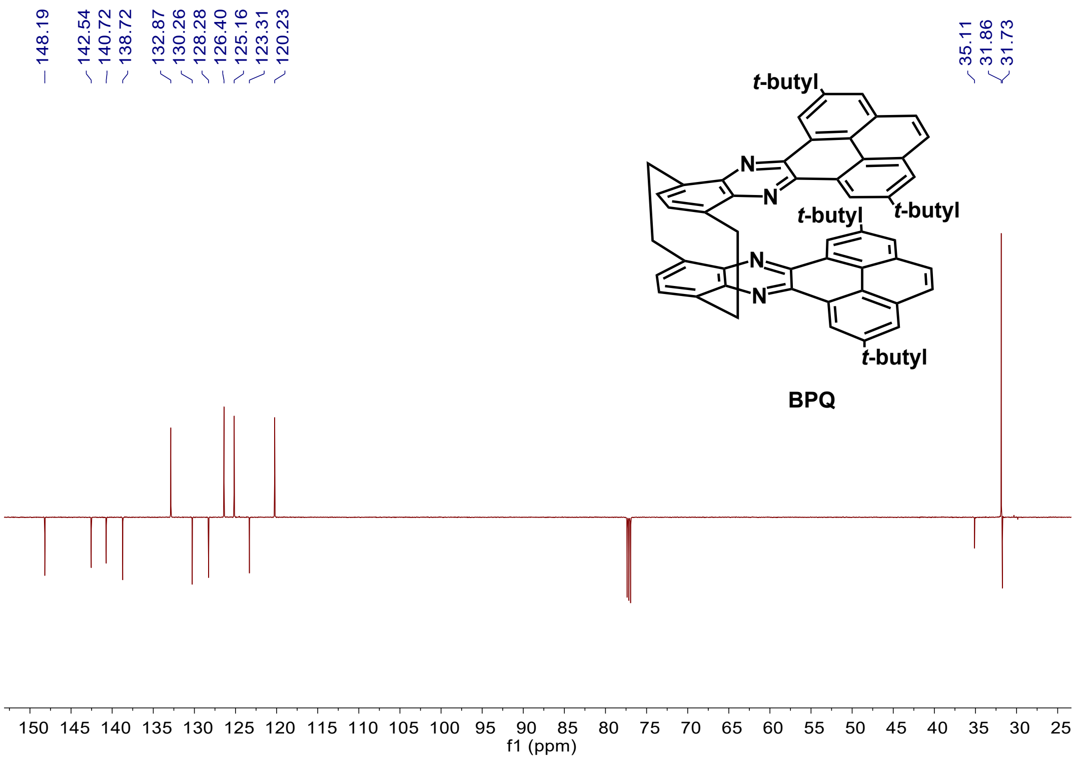

Fig. S8 The attached proton test (APT) ^{13}C NMR spectrum of compound BPQ in $CDCl_3$ on 600 MHz.

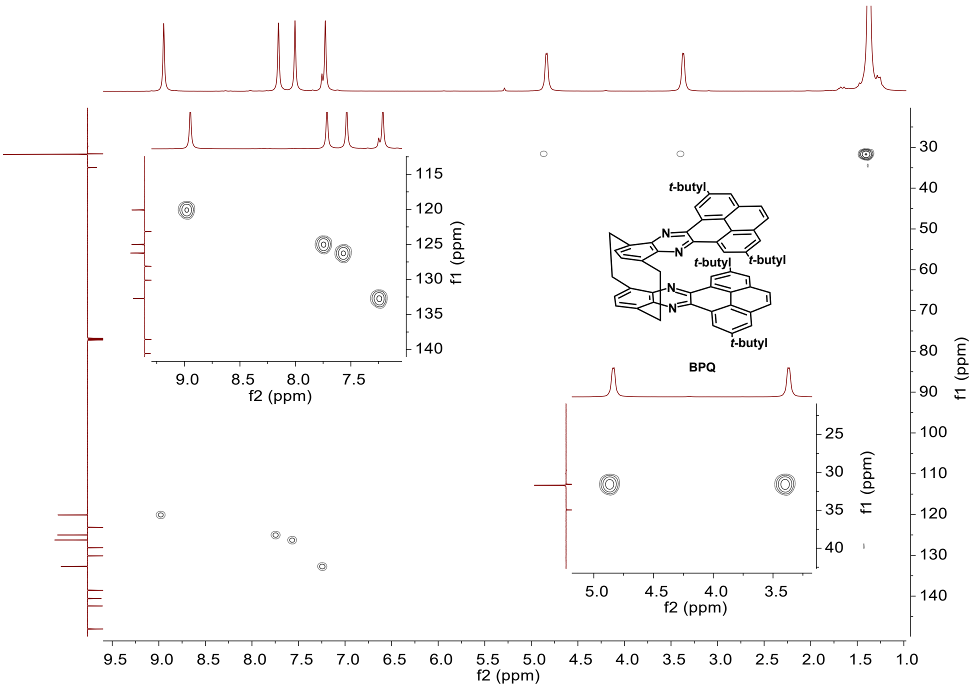

Fig. S9 The 2D ^{1}H–^{13}C heteronuclear single quantum coherence (HSQC) NMR spectrum of BPQ in $CDCl_3$ on 600 MHz.

HRMS Spectra

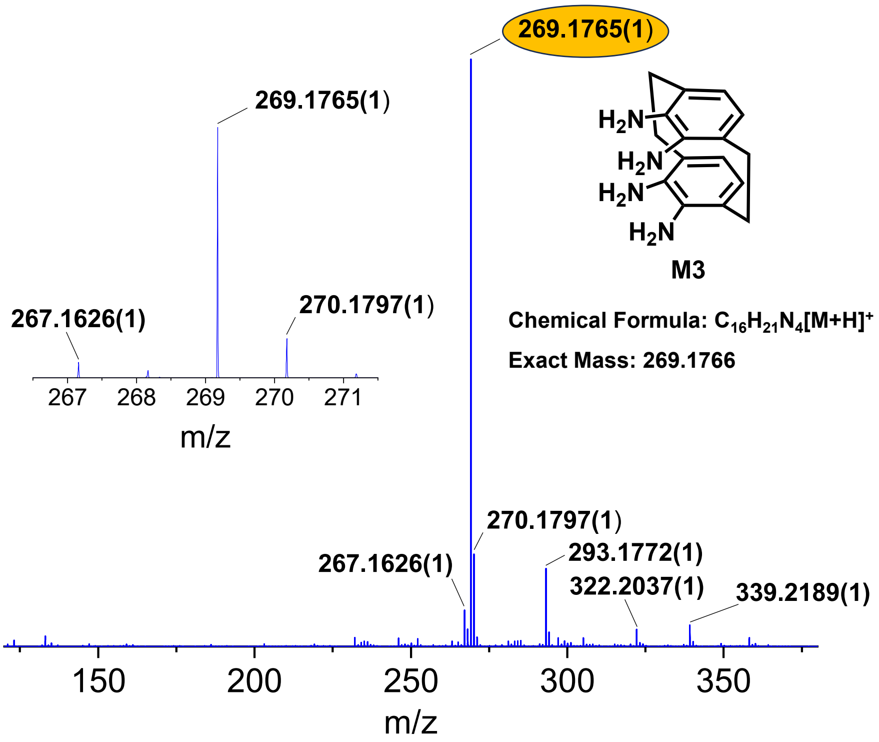

Fig. S10 High-resolution mass spectrum of M3($[M+H]^+$).

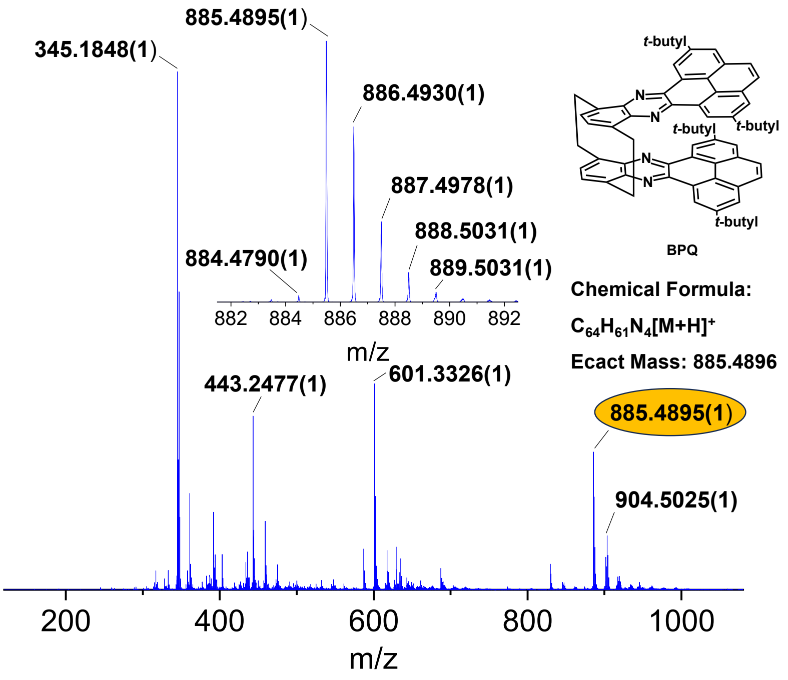

Fig. S11 High-resolution mass spectrum of BPQ($[M+H]^+$).

Electrochemical Measurement

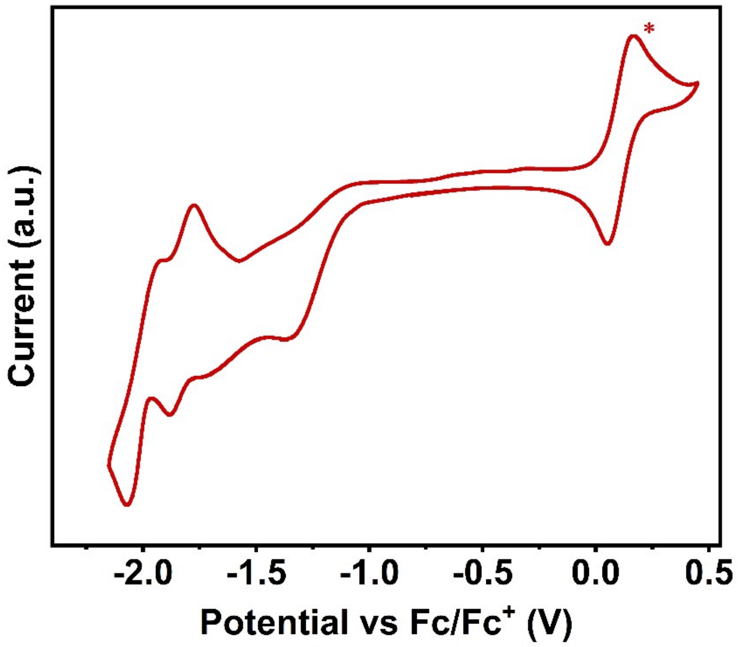

Fig. S12 Cyclic voltammogram of BPQ in THF with ferrocene (ferrocene peaks are indicated by red star and occur at positive potential) as an internal standard, the energy of Fc/Fc^+ was assumed as −4.8 eV relative to vacuum.

Single Crystal X-Ray Analysis

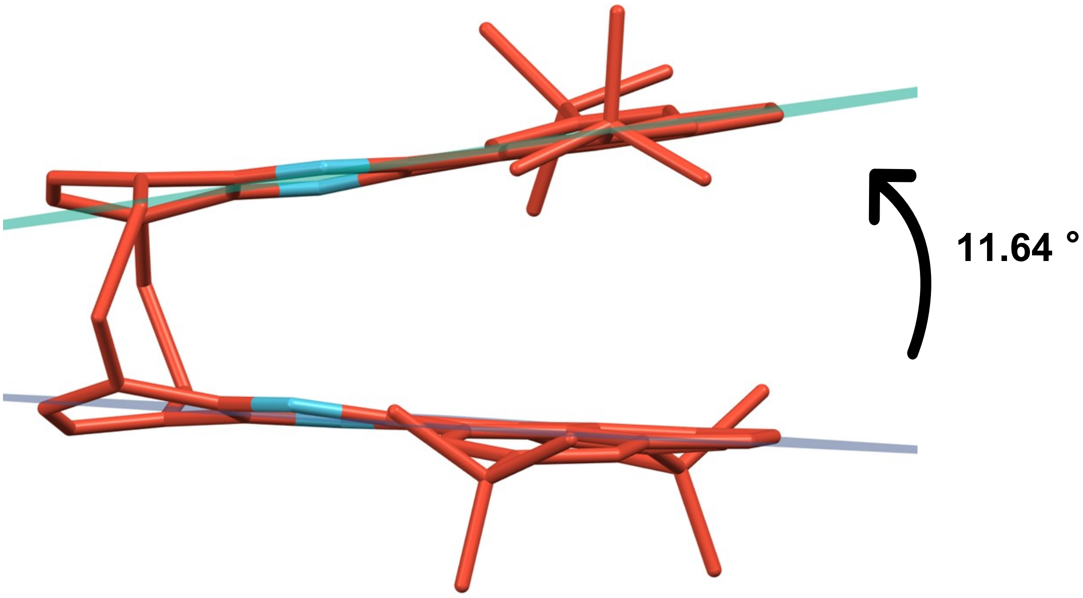

Fig. S13 Dihedral angle of BPQ.

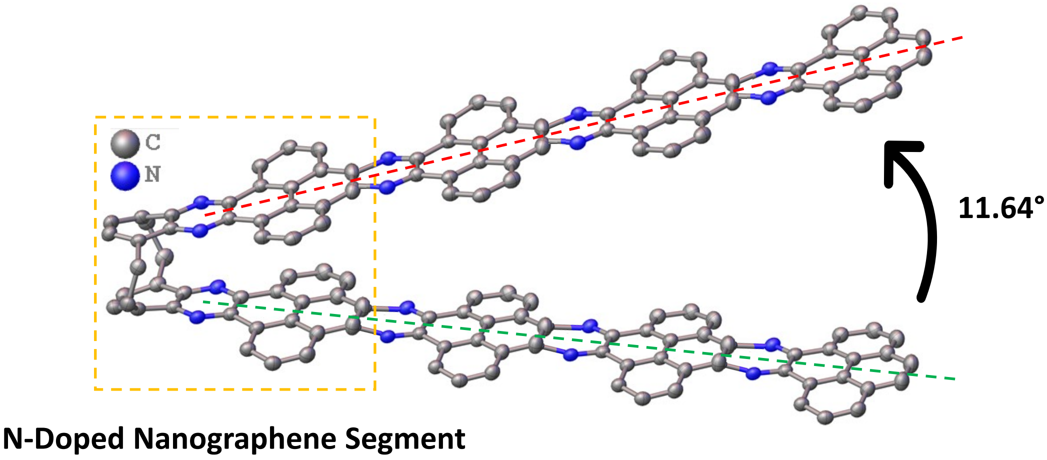

Fig. S14 BPQ can be described as a segment of N-doped nanographene.

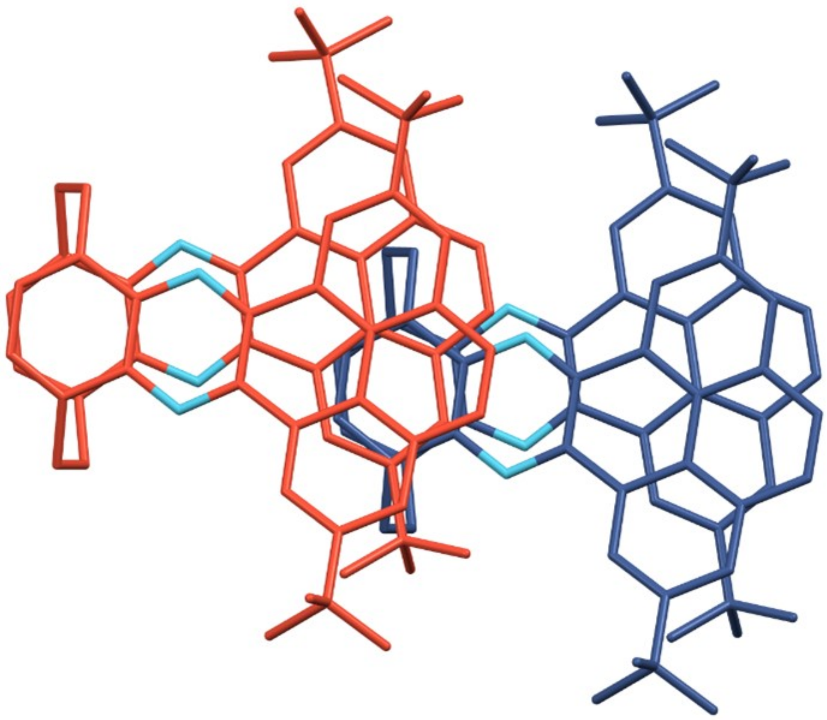

Fig. S15 The π-π overlap of BPQ.

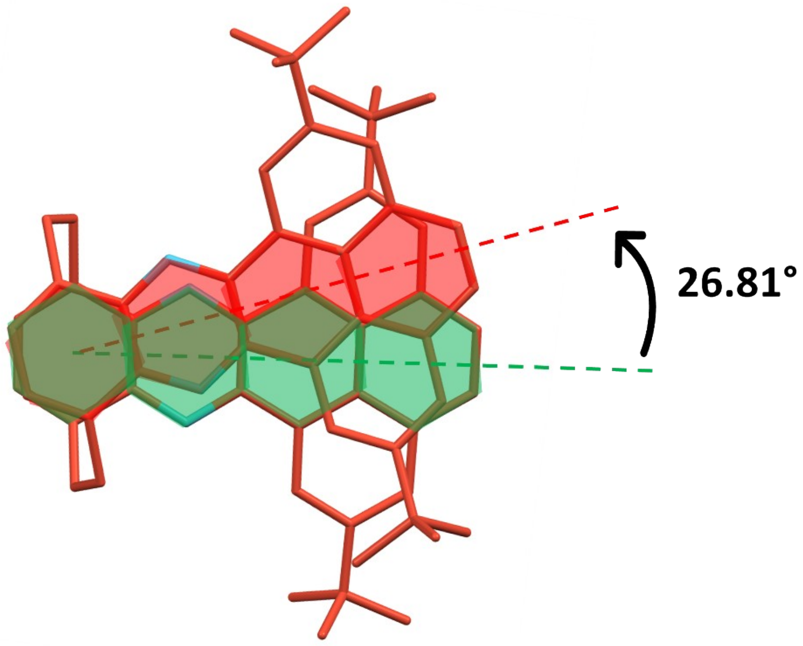

Fig. S16 The displacement of the upper and lower acene frameworks containing the pyrene skeleton.

Table S1. Crystal data and structure refinement for BPQ.

Identification code	**BPQ**
CCDC number	2322933
Empirical formula	$C_{64}H_{60}N_4$
Formula weight	885.16
Temperature/K	194.00
Crystal system	monoclinic
Space group	C2/c
a/Å	36.4386(8)
b/Å	9.7054(3)
c/Å	30.3886(7)
α/°	90
β/°	110.4310(10)
γ/°	90
Volume/Å^3	10070.9(5)
Z	8
ρ_{calc}g/cm^3	1.168
μ/mm^{-1}	0.515
F(000)	3776.0
Crystal size/mm^3	0.13 × 0.12 × 0.1
Radiation	CuKα (λ = 1.54178)
2Θ range for data collection/°	6.208 to 159.886
Index ranges	$-43 \leq h \leq 45$, $-12 \leq k \leq 12$, $-37 \leq l \leq 23$
Reflections collected	50391
Independent reflections	10415 [R_{int} = 0.0811, R_{sigma} = 0.0545]
Data/restraints/parameters	10415/144/688
Goodness-of-fit on F^2	1.075
Final R indexes [I>=2σ (I)]	R_1 = 0.0711, wR_2 = 0.1930
Final R indexes [all data]	R_1 = 0.1057, wR_2 = 0.2310
Largest diff. peak/hole / e Å^{-3}	0.34/-0.37

References

1. M. J. Frisch, et al. Gaussian 16, Revision A.03; Gaussian, Inc.: Wallingford CT, 2016.
2. A. D. Becke, *J. Chem. Phys.*, 1993, **98**, 1372.
3. C. T. Lee, W. T. Yang and R. G. Parr, *Phys. Rev. B*, 1988, **37**, 785-789.
4. O. V. Dolomanov, L. J. Bourhis, R. J. Gildea, J. A. K. Howard and H. Puschmann, *J. Appl. Crystallogr.*, 2009, **42**, 339-341.

5. G. M. Sheldrick, *Acta Crystallogr. C Struct. Chem.*, 2015, C71, 3-8.
6. J. Hu, D. Zhang and F. W. Harris, *J. Org. Chem.*, 2005, **70**, 707-708.
7. M. Watanabe, K. Goto, M. Fujitsuka, S. Tojo, T. Majima and T. Shinmyozu, *Bull. Chem. Soc. Jpn.*, 2010, **83**, 1155-1161.
